# Verses

**Published:** 1906-12

**Authors:** 


### VERSES.

"Enough of science and of art;
  Close up these barren leaves.
Come forth! and bring with you a heart
  That watches, and receives."

—Good Health.

### MAN AND THE VINE.

"God made man
Frail as a bubble;
God made love.
Love made trouble;
God made vine.
Was it a sin
That man made wine
To drown trouble in?"

### TO WOMEN.

"Here's to women who are tender,
Here's to women who are slender,
Here's to women who are large and fat and red;
Here's to women who are married,
Here's to women who have tarried,
Here's to women who are speechless—but they're dead."

### AN IMPATIENT PATIENT.

A patient knocked at a dentist's door,
Who had slept little the night before,
The pain was so great he was ready to roar,
The dentist compelled him to sit near the floor.
With drilling machine he commenced to bore,
Till the nerve was touched up, when, awfully sore,
The patient refused to stand an more.
It is sometimes unpleasant to tell the truth,
He lost that patient, but saved the tooth.

—Dental Surgeon.

### GOING HUNTING.

De Possum een er gum stump,
De Coon een er hollow,
De squrl on er hickory lim,
De Rabbit on de fallow.
De Doves am er drovin now,
De Patridge scratchin groun
De Robins am cumin Souf
Ole fiel Larks am flyin roun.
So cum erlong, mer yaller dog,
Wid ketridges an gun,
We'll go to de woods and fiels,
Ter hab sum game an fun.

—B. H. Teagne.

